# Presenting clinical characteristics of open globe injuries in ocular trauma: baseline analysis of cases in the ASCOT national clinical trial

**DOI:** 10.1038/s41433-022-02206-z

**Published:** 2022-09-14

**Authors:** Suzie Cro, Giles Partington, Victoria R. Cornelius, Philip J. Banerjee, Tapiwa Margaret Zvobgo, Edward J. Casswell, Syed Shahid, Catey Bunce, Elizabeth Robertson, Caroline Murphy, Joanna Kelly, David G. Charteris

**Affiliations:** 1 Imperial Clinical Trials Unit, Imperial College London, London, UK; 2 Frimley Health NHS Foundation Trust, London, UK; 3 Moorfields Eye Hospital NHS Foundation Trust, London, UK; 4 The Royal Marsden NHS Foundation Trust, London, UK; 5 King’s Clinical Trials Unit at Kings Health Partners, King’s College London, London, UK

**Keywords:** Eye manifestations, Risk factors

## Abstract

**Background/Objectives:**

The Adjunctive Steroid Combination in Ocular Trauma (ASCOT) trial is a unique pragmatic, multi-centre, patient and assessor masked, randomised controlled trial. We evaluate the clinical characteristics and pathology of this large trial cohort of patients with open globe injuries undergoing vitreoretinal surgery, including the associations between patient characteristics and their baseline vision.

**Subjects/Methods:**

We (i) summarise demographics, injury history and ocular history of the 280 participants recruited into the ASCOT trial using descriptive statistics; (ii) analyse the national and seasonal variation across England and Scotland in these participant characteristics; and (iii) explore the associations between participant demographic, trauma history, ocular history and presenting baseline visual acuity (measured using the Early Treatment Diabetic Retinopathy Study, ETDRS) using multivariable regression analyses.

**Results:**

The majority of participants with open globe penetrating injuries were of white ethnicity (233, 84%), male (246, 88%), with a median age of 43 years (IQR 30–55 years). There was considerable variability in presenting visual acuity with 75% unable to read any letters on the ETDRS chart, whilst the median ETDRS letter score was 58 (IQR 24–80) for those who could read ≥1 letter. The most common causes of injury were workplace related (31%) or interpersonal violence (24%). Previous eye surgery, visual axis corneal scar, lens status, hyphaemia and vitreous haemorrhaging were found to be associated with presenting vision as measured by the ETDRS chart.

**Conclusion:**

The ASCOT trial provides valuable insights into the spectrum of pathology of patients with open globe eye injuries undergoing vitreoretinal surgery. The identified causes of injury and clinical presentation of the cases will help in training and resource planning to deal with these often challenging surgical cases.

**Trial registration:**

EudraCT No. 014-002193-37. HTA Project 12/35/64.

## Introduction

Ocular trauma is a leading cause of monocular visual impairment and monocular blindness worldwide [1]. Globally, 55 million people experience serious ocular trauma every year [2]. It has been estimated that worldwide 1.6 million people are blind as a result of ocular trauma, with 2.3 million having bilateral low vision and 19 million with unilateral vision [2]. The prevalence of ocular trauma has been shown to vary considerably by country, gender, age and socio-economic status [3–6]. Blindness from ocular trauma is a serious health problem that has extensive psychological impacts on patients and their relatives, and major socio-economic costs [7].

Frequently the posterior segment of the eye, comprising the vitreous humour, retina, choroid, and optic nerve, is affected by injuries that result in visual loss and to prevent severe loss of vision, posterior segment (vitreoretinal) surgery is necessary. In a one-year population-based prospective study of ocular trauma in Scotland, among cases of serious eye injury blinding outcomes occurred in 26% [8]. Whilst surgical techniques have improved, outcomes remain unsatisfactory. This is mainly due to the development of the intraocular scarring response proliferative vitreoretinopathy (PVR). PVR is the main cause of visual loss in eyes with open globe injuries (defined as a full thickness wound of the eye wall) and is estimated to occur in 10–45% of all open globe injuries [9–12].

Experimental studies have suggested that steroid (triamcinolone acetonide, TA) treatment can reduce the severity of PVR [13]. The primary aim of the Adjunctive Steroid Combination in Ocular Trauma (ASCOT) trial is to assess whether, adjunctive intravitreal and sub-Tenon’s triamcinolone acetonide given at the time of surgery improves visual acuity at 6 months compared with standard surgery. ASCOT is a phase 3 randomised controlled trial which recruited a total of 280 eligible patients nationally across 27 sites in England and Scotland [14, 15]. The trial began recruitment in December 2014 and completed recruitment in March 2020; final participant follow-up was completed in September 2020.

ASCOT is the only large scale prospective multi-centre national trial to have been undertaken on traumatic open globe injuries and therefore, for the first time, provides a unique opportunity to evaluate the detailed clinical characteristics of a large cohort of patients with open globe injuries. A previous national study of serious eye trauma in Scotland reported an incidence of 1.96/100,000 but did not report any detail on clinical presentations [8]. The aims of this analysis are to evaluate the clinical characteristics and pathology of this large trial cohort of patients with open globe injuries undergoing vitreoretinal surgery, including the associations between patient characteristics and their baseline vision. Specific objectives are (1) to describe demographics, injury history and ocular history of participants recruited into the ASCOT trial, (2) to analyse the national and seasonal variation across England and Scotland in these participant characteristics, and (3) to explore associations between participant demographic, trauma history, ocular history and presenting baseline visual acuity.

## Subjects and methods

Full details of the ASCOT study design have been published [14]. Briefly, ASCOT is a pragmatic, multi-centre, double-masked, randomised controlled trial. The study population included adult patients (aged 18 years or over) with a full thickness, open globe injury undergoing vitrectomy with the ability to give informed consent. Exclusion criteria were patients with pre-existing uncontrolled uveitis, previous steroid-induced glaucoma, pregnant or breastfeeding females, those with previous known adverse reactions to triamcinolone, inability to attend regular follow-up, current or planned use of corticosteroid use of a dose above physiological levels. All penetrating injuries attending eye emergency clinics were screened and patients were recruited at vitreoretinal outpatient or emergency clinics at 27 participating vitreoretinal surgical centres throughout the UK. Eligible participants were randomised (1:1) to standard surgery plus adjunctive triamcinolone acetonide or standard surgery alone for the operated eye. Operating surgeons were masked until the end of surgery; participants and study investigators were masked to treatment allocation throughout. For patients with bilateral injuries, data is reported for the worse injured eye, which was considered as the study eye for randomisation and operated on according to randomisation (the better eye received standard treatment). The ASCOT study protocol was approved by the Central London NHS Research Ethics Committee (14/LO/1428).

### Data collection and outcomes

In this report, we analyse participant baseline demographics, trauma history and ocular history including baseline biomicroscopic ocular exam results prior to surgery. The baseline eye exam included the Early Treatment Diabetic Retinopathy Study (ETDRS) letter score measured using the standard validated ETDRS chart at a starting distance of 4 m in the eye operated on. If 20 or more letters were read at 4 m, 30 was added to the total letter score. If less than 20 letters were read correctly at 4 m then the distance was moved to 1 m and the number of letters read at 1 m formed the total score. If no letters were seen at 1 m then the score is 0 and test for Counting Fingers, Hand Movements and Light Perception.

### Statistical analysis

The target sample size for ASCOT was 300 patients (150 per arm) over a 3-and-a-half-year recruitment period. As actual recruitment rate was slower than projected, the recruitment period was extended to a total of 75 months; 280 eligible patients were recruited and all are included within this analysis.

Baseline characteristics are summarised as mean (standard deviation—SD) for approximately normally distributed continuous variables or median (Interquartile range—IQR) where skewed. Categorical variables are summarised by frequencies and percentages. Descriptive data summaries are based on observations only and the number of missing observations are reported. The Pearson’s chi-squared test was used to assess seasonal variation in the injury numbers by month, and cause of injury.

To explore the associations between baseline visual acuity (measured using an ETDRS chart at a starting distance of 4 m) and participant characteristics of interest, multivariable regression analyses were conducted. The clinically important characteristics of interest, informed by previous studies and study team clinical opinions were: previous primary repair, trauma classification, extent of trauma, previous eye surgery, macular disease, other historical ocular conditions, visual axial corneal scarring, hyphaemia, lens state, vitreous haemorrhage, endophthalmitis, retinal status and PVR. Given the high distribution of participants with an ETDRS score of 0 letters indicating very low/no vision, a zero-inflated Negative Binomial model was used to explore associations. This model incorporates two parts; (i) a logistic regression model that models the probability of zero/very low vision (ETDRS = 0) and (ii) a Negative Binomial model that models the amount of vision as measured by the ETDRS letter count score. The Negative Binomial model (part ii) included all the clinically important variables of interest. The logistic regression model (part i) used a subset of the clinically important variables that were first identified to be associated with zero vision (see Supplementary file 1, Table S3).

Retinal detachment at baseline and associations between the clinically important characteristics of interest were explored in an additional analysis using a logistic regression model. Estimates from the logistic regression models are summarised as Odds Ratios (OR). Estimates from the zero-inflated Negative binomial model are presented as Incidence Rate Ratios (IRR) which represent the relative risk of a higher ETDRS score for the associated variable. Estimates are accompanied with 95% confidence intervals. A *p* value <0.20 was used to indicate a potential association and a result of interest. As exploration of missing data patterns revealed <10% of the sample would be excluded due to missing data, the analysis included all participants with complete data under the assumption that any missing data were missing completely at random. All statistical analysis was performed using Stata/IC version 15.0 or above.

## Results

### Baseline characteristics

Table 1 summarises the baseline demographics, trauma history and ocular history for the study cohort of 280 patients. The majority of participants were of white ethnicity (233, 84%), male (246, 88%), with a median age of 43 years (IQR 30–55 years). The median age of the females was 63.0 years (IQR 44.6–75.6) and for the males was 42.2 years (IQR 29.9–3.3). The causes of the ocular trauma are indicated in Table 1; 14% (38) were classified as “other injury” and these are listed in Supplementary Table S1.

**Table 1:** Baseline demographics, trauma history and ocular history.

Variable [*n* missing]	*N*	%
Gender (*n*, %) [*n* = 0]		
Female	34	12%
Male	246	88%
Region (*n*, %) [*n* = 0]		
London	148	53%
South England	55	20%
Midlands & North England	70	25%
Scotland	7	3%
Ethnicity (*n*, %) [*n* = 0]		
White	233	83%
Black	20	7%
Asian	18	6%
Other	6	2%
Mixed	3	1%
Eye injured (*n*, %) [*n* = 0]		
Right	137	49%
Left	138	49%
Both	5	2%
How was the eye injured (*n*, %) [*n* = 0]		
Workplace incident	88	31%
Road traffic accident	11	4%
Interpersonal violence	66	24%
Sports injury	10	4%
Other injury^a^	37	13%
Other domestic^b^	21	8%
Domestic gardening	8	3%
Domestic DIY	13	5%
Iatrogenic	3	1%
Fall	23	8%
Previous primary repair (*n*, %) [*n* = 1]	205	73%
Severity of trauma: classification (*n*, %) [*n* = 0]		
Rupture	113	40%
Penetrating	103	37%
Perforating	11	4%
IOFB	53	19%
Severity of trauma: extent (*n*, %) [*n* = 2]		
Cornea	95	34%
Scleral anterior to muscle insertion	110	40%
Scleral posterior to muscle insertion	72	26%
Severity of trauma: RAPD present? - Yes (*n*, %) [*n* = 2]	43	15%
Not documented	140	50%
Glaucoma (*n*, %) [*n* = 1]	4	1%
Previous eye surgery (study eye) (*n*, %) [*n* = 0]	149	53%
Macular disease (*n*, %) [*n* = 1]	1	0%
Other historic ocular conditions (*n*, %) [*n* = 2]	29	10%
Visual axis corneal scar (*n*, %) [*n* = 0]	72	26%
Uveitis (n, %) [*n* = 0]	52	19%
Hyphaemia level (*n*, %) [*n* = 0]		
No hyphaemia	186	66%
<50%	50	18%
>50%	44	16%
Iris state (*n*, %) [*n* = 4]		
Normal	111	40%
Incomplete	135	49%
Incarcerated	30	11%
Lens state (*n*, %) [*n* = 3]		
Clear	70	25%
Cataract	96	35%
ACIOL	2	1%
PCIOL	20	7%
Aphakic	89	32%
Vitreous haemorrhage (*n*, %) [*n* = 4]	182	66%
If vitreous haemorrhage present (*n*, %) [*n* = 0]		
No fundal view	139	76%
VH with fundus visible	43	24%
Endophthalmitis present (*n*, %) [*n* = 1]	5	2%
Retinal status (*n*, %) [*n* = 0]		
Attached	137	49%
TRD	38	14%
RRD	105	38%
Was the fovea off - Yes (*n*, %) [*n* = 0]	85	59%
Splitting	1	1%
PVR present (*n*, %) [*n* = 1]	67	24%
Age (years) [*n* = 0]		
Median, IQR	43.5	(30.9, 55.8)
Time from injury to surgery (days) [*n* = 0]		
Median, IQR	20	(7, 61)
IOP in study eye [*n* = 26]		
Median, IQR (mmHg)	11	(8,15)
Categorised IOP [*n* = 26]		
Low IOP (<6)	39	15%
Normal IOP (6 ≤ *IOP* ≤ 22)	195	77%
High IOP (>22)	20	8%
ETDRS in eye operated on (total score) [*n* = 0]		
Median, IQR	0	(0.0, 1.0)
Min, Max		(0.0, 100.0)
ETDRS in eye operated on [*n* = 0]		
0 (none/very low vision)	209	75%
>0	71	25%
Where ETDRS = 0, level of vision in eye operated on [*n* = 0]		
Count fingers	19	9%
Hand movement	114	55%
Perception of light	71	34%
No perception of light	5	2%
Where ETDRS > 0 in eye operated on [*n* = 0]		
Mean, SD	53	29.1
Median, IQR	58	(24, 80)
Min, Max		(1, 100)

Percentages have been rounded throughout to nearest 1 d.p. so may not always sum to 100%.

*IOFB* intraocular foreign body, *TRD*  tractional retinal detachment, *RRD*  rhegmatogenous retinal detachment, *VH* vitreous haemorrhage, *RAPD*  relative afferent pupillary defect, *PVR*  proliferative vitreoretinopathy, *IOP* intraocular pressure, *ACIOL* anterior chamber intraocular lens, *PCIOL* Posterior chamber intraocular lens, *IQR* Interquartile range, *ETDRS* Early Treatment Diabetic Retinopathy Study.

^a^Other Injury includes any injuries that do not fall into any other category (e.g. freak accidents/childhood play), or where not enough information was given to categorise. For a full listing of other injury causes see supplementary file.

^b^Other Domestic includes all other injuries within a domestic setting not covered by Domestic Gardening, Domestic DIY or Falls. For a full listing of other injury causes see supplementary file.

The majority of participants (209, 75%) had a score of zero on the ETDRS chart meaning they presented with very low vision of counting fingers or worse. The median ETDRS score among those with an ETDRS score greater than zero (71, 25%), was 58 letters (IQR 24–80) demonstrating high variability in presenting vision.

### Regional variability

The majority (148, 53%) of ASCOT participants were recruited from hospitals in London, a quarter came from the Midlands & Northern England (70, 25%), 55 (20%) were recruited in Southern England and 7 from Scotland (3%). London had a higher proportion of Black, Asian and Minority Ethnic (BAME) participants (24%) than any other region (Southern England: 4%, Midlands and North England: 11% and Scotland: 0%). This variation reflects the regional ethnic diversity in the wider population, at the most recent 2011 census 40% of London residents identified as BAME versus 8% in Southern England, 11% in the Midlands and North England and 4% in Scotland [16, 17]. Supplementary Table S2 summarises baseline characteristics by region (London, Southern England, Midlands & Northern England, and Scotland).

There was little difference between the causes of injury across the regions. In London, nearly half (70, 47%) of all injuries were categorised as penetrating, with 46 (31%) as rupture. However, all other regions had substantially higher amounts of ruptures than penetrations. Similarly, injuries in London were more frequently to zone 1 of the eye (corneal injury) than to zone 2 (scleral anterior) (39% vs 35% [58 vs 52]), whereas the reverse was true in other regions. There was a higher history of ocular co-morbidities for patients in London (21, 14%) and Midlands & North England (7, 10%).

More than 25% of patients in London and Scotland had an ETDRS score above zero (zero indicating very low vision of counting fingers or worse).

### Seasonal variation

**Fig. 1:**
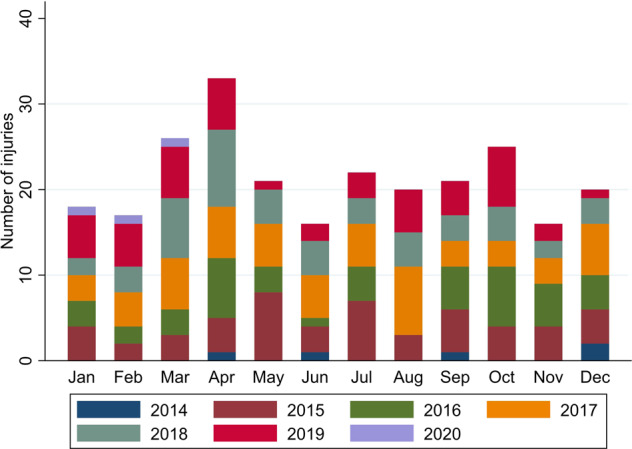
Seasonal variation in the number of ocular injuries. Number of injuries shown by year (represented by different colours) and month.

The dates of ocular injuries were investigated for seasonal trends. Only injuries from 2014 were included as the date of injury was not collected accurately prior to this time, resulting in 255 participants included within this analysis. The most common month for injury was April and injuries in April were consistently high across each year of recruitment. January, February, June and November had slightly fewer injuries occurring than in other months (see Fig. 1). There was however no evidence to suggest significant variation in the number of injuries by month beyond that expected by chance (*p* = 0.293, *χ*^2^ = 13, 11 degrees of freedom).

**Fig. 2:**
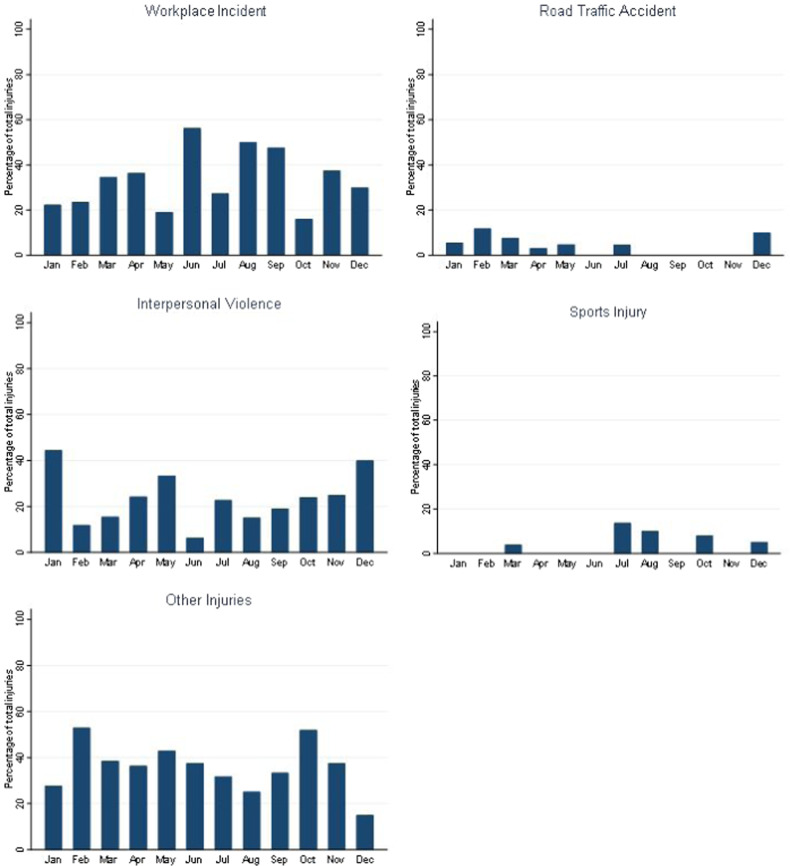
Causes of ocular injuries. Proportion of injuries (as a percentage) by cause of injury (workplace, road traffic accident, interpersonal violence, sports injury and other) and month of injury.

Workplace incidents and interpersonal violence accounted for the majority of ocular injuries and there were fluctuations in the months these injuries occurred (see Fig. 2A–E). Interpersonal violence injuries peaked in December to January and April to May, with lows in February and June. Workplace injuries occurred most often in April, closely followed by August and September, with lulls in January, February, May and October. There was no evidence to suggest cause of injury differed significantly across month of injury (*p* = 0.534, *χ*^2^ = 97.1, 99 degrees of freedom).

### Associations between trauma and ocular history and presenting vision

Table 2 displays the regression model coefficients and 95% CI’s for the multivariable regression analyses that explore the associations between patient history, presenting clinical characteristics and presenting vision (ETDRS).

**Table 2:** Associations with presenting vision as measured by the ETDRS.

Variable	Zero-inflated negative binomial			Zero-Inflated Logistic Regression (ETDRS = 0)		
	IRR^a^	95% CI	*p* value	OR^b^	95% CI	*p* value
Previous primary repair						
No (reference)						
Yes	1.1	0.63, 1.91	0.742			
Classification						
Rupture (reference)			0.801			
Penetration	0.92	0.60, 1.43				
Perforation	0.54	0.07, 4.10				
IOFB	1.13	0.63, 2.04				
Extension zone						
Cornea (reference)			0.274			
Scleral–anterior to muscle insertion	1.37	0.92, 2.04				
Scleral–posterior to muscle insertion	1.13	0.67, 1.92				
Previous eye surgery (eye operating on)						
No (reference)			0.001			
Yes	0.41	0.25, 0.69				
Macular disease						
No (reference)			0.98			
Yes	0	0				
Other eye history						
No (reference)			0.249			
Yes	0.74	0.45, 1.23				
Visual axis corneal scar						
No (reference)			0.048			
Yes	1.59	1.0, 2.51		2.33	0.95, 5.67	0.063
Hyphaema						
None (reference)			0.469			0.018
<50%	1.14	0.60, 2.17		5.24	1.34, 20.54	
>50%	0.48	0.14, 1.64		6.55	0.78, 55.24	
Lens status						
Clear (reference)			0.128			
Cataract	0.74	0.48, 1.14				
ACIOL	2.05	0.75, 5.60				
PCIOL	1.11	0.60, 2.06				
Aphakic	0.69	0.41, 1.16				
Vitreous haemorrhage						
None (reference)			0.189			<0.001
VH with no fundal view	0.61	0.33, 1.15		8.45	3.07, 23.20	
VH with fundus	1.11	0.79, 1.57		0.29	0.11, 0.76	
Endophtamlitis						
No (reference)						
Yes	1.4	0.39, 5.11	0.606			
Retinal status						
Attached (reference)			0.305			
TRD	0.64	0.27, 1.53				
RRD	1.26	0.85, 1.87				
Proliferative vitreoretinopathy (PVR)						
No (reference)						
Yes	1.38	0.71, 2.69	0.338	7.2	2.30, 22.61	0.001

*ACIOL* anterior chamber itraocular lens, *PCIOL* posterior chamber introcular lens, *IOFB* intraocular foreign body, TRD tractional retinal detachment, *RRD* rhegmatogenous retinal detachment. *VH* vitreous haemorrhage, *PVR* proliferative vitreoretinopathy, *ETDRS* Early Treatment Diabetic Retinopathy Study.

^a^IRR (Incidence rate ratio) represents the relative risk of a higher ETDRS score for each associated variable relative to the reference group for that variable.

^b^OR (Odds ratio) represents the odds of ETRDS score ≤0, for the associated variable relative to the reference group for that variable.

 Factors associated with  zero vision (an ETDRS score of 0 letters indicating very low/no vision) were visual axis corneal scar (*p* = 0.063), hyphaema (*p* = 0.018), vitreous haemorrhage (*p* < 0.001) and PVR (*p* = 0.001) (see Table 2), indicating that if the trauma causes these then very poor vision at presentation is more likely. Having hyphaema greatly increased the odds of zero/very low vision for both those with <50% hyphaema OR = 5.24 (95% CI 1.34, 20.5) and those with >50% hyphaema OR = 6.55 (95% CI 0.78, 55.2) relative to those without hyphaema. Vitreous haemorrhage with no fundal view had much higher odds of zero/very low vision in comparison to without visual haemorrhage OR = 8.45 (95% CI 3.07, 23.2), whereas visual haemorrhage with fundus had much lower odds of zero/very low vision OR = 0.29 (95% CI 0.11, 0.76) compared to without visual haemorrhage.

For individuals who could read at least one letter on the ETDRS chart (ETDRS score >0), previous eye surgery (*p* = 0.001), visual axis corneal scar (*p* = 0.048), lens status (*p* = 0.128) and vitreous haemorrhaging (*p* = 0.189) were identified to be associated with presenting ETDRS score. The expected ETDRS score for patients who had had previous eye surgery was decreased by a factor of 0.41 times (95% CI 0.25, 0.69) relative to no previous surgery among participants with ETDRS > 0. In comparison to a clear lens, having an anterior chamber intraocular lens (ACIOL) was associated with better vision, IRR = 2.05 (95% CI 0.75, 5.60, *p* = 0.159), whereas those with cataracts IRR = 0.74 (95% CI 0.48, 1.14), and aphakic IRR = 0.69 (95% CI 0.42, 1.16,) had lower ETDRS scores among participants with ETDRS > 0. The ETDRS score with vitreous haemorrhaging and no fundal view was 0.61 times (95% CI 0.33, 1.15) with no vitreous haemorrhage among participants with ETDRS > 0. The ETDRS score with visual axis corneal scar was 1.59 times higher (95% CI 1.00, 2.51) in comparison to without among participants with ETDRS > 0—however, those with visual axis corneal scare were more likely to have no vision at presentation than those without (although not statistically significantly so).

Associations with retinal detachment are shown in Table 3. Hyphaema was shown to be strongly associated with retinal detachment (*p* = 0.033); over 50% hyphaema was associated with higher odds of retinal detachment relative to none, OR = 3.29 (95% CI 1.32, 8.16). The location of injury also had an association (*p* = 0.015) with retinal detachment with injuries to scleral-posterior to muscle insertion having higher odds of retinal detachment 3.43 (95% CI 1.44, 8.19) relative to injuries extending to the cornea only.

**Table 3:** Associations with retinal detachment.

Variable	OR^a^	95% CI	*p* value
Previous primary repair			
No (reference)			0.235
Yes	1.66	0.72, 3.86	
Classification			
Rupture (reference)			0.966
Penetration	0.9	0.47, 1.74	
Perforation	0.8	0.19, 3.35	
IOFB	1.08	0.40, 2.93	
Extension zone			
Cornea (reference)			0.015
Scleral–anterior to muscle insertion	1.45	0.71, 2.98	
Scleral–posterior to muscle insertion	3.43	1.44, 8.19	
Previous eye surgery (study eye)			
No (reference)			0.096
Yes	1.75	0.91, 3.37	
Macular disease			
No (reference)			
Yes	N/A		
Other eye history			
No (reference)			0.907
Yes	1.06	0.42, 2.68	
Visual axis corneal scar			
No (reference)			0.943
Yes	1.02	0.52, 2.01	
Hyphaema			
None (reference)			0.033
<50%	1.83	0.82, 4.08	
>50%	3.29	1.32, 8.16	
Lens status			
Clear (reference)			0.715
Cataract	1.1	0.51, 2.40	
ACIOL	N/A		
PCIOL	0.69	0.19, 2.47	
Aphakic	1.31	0.57, 3.01	
Vitreous haemorrhage			
None (reference)			0.117
VH with no fundal view	0.47	0.22, 1.00	
VH with fundus	0.5	0.20, 1.23	
Endophthamitis			
No (reference)			
Yes	0.43	0.04, 4.81	0.495

*ACIOL* anterior chamber itraocular lens, *PCIOL* Posterior chamber introcular lens, *IOFB* Intraocular foreign body. *TRD* tractional retinal detachment, *RRD* rhegmatogenous retinal detachment, *VH* vitreous haemorrhage.

^a^OR (odds ratio) represents the odds of ETRDS score >0, for the associated variable relative to the reference group for that variable.

## Discussion

The ASCOT study was a prospective randomised controlled trial designed to test the efficacy of intraocular and periocular triamcinolone given at the time of vitrectomy surgery in cases of open globe injury. It recruited nationally in the UK from 27 sites. The baseline data therefore represents the cases considered suitable for inclusion by the principal investigators at the individual sites and is not a comprehensive epidemiological study on open globe injuries. Nevertheless, it provides detailed data on a large cohort of patients undergoing vitrectomy surgery.

The study population were overwhelmingly (88%) male, consistent with other analyses of eye trauma epidemiology [8, 18, 19].The median age of the study population was 43 demonstrating the difference between trauma patients and the majority of other patients undergoing intraocular surgery. There was considerable variability in presenting visual acuity. Five patients had no perception of light (NPL) vision at baseline indicating that amongst UK vitreoretinal surgeons there is some willingness to operate on patients with very limited vision. Internationally other reports have documented patients with NPL vision following penetrating trauma undergoing vitreoretinal surgery although the prognosis for these cases remains very poor [20].

There was a broad range of causal mechanisms of the injury in the study cases. The most common setting was the workplace (31%)followed by a significant number of the injuries (24%) due to interpersonal violence. In the UK, regulations relating to eye protection in the workplace are included in 1992 legislation on personal protective equipment at work, which came into effect in 1993 (recently updated in 2022 to also cover more causal employment relationships in addition to contracted employees) [21, 22]. The regulations clarify how suitable eye care should be provided to employees who may be exposed to a risk. Despite these regulations, the results of this study emphasise the need for further efforts to improve eye protection in the working environment. The majority of cases presented with a penetration (37%) or a globe rupture (40%) with smaller numbers having a perforation (4%) or an intraocular foreign body (19%).

The baseline clinical characteristics provide a useful insight into the range of pathology likely to be encountered by vitreoretinal surgeons. The initial view of the posterior segment is often compromised with 26% presenting with a visual axis corneal scar and 34% a hyphaema. Anterior segment pathology is likely with only 25% having a clear lens (35% had a cataract which may require combined surgery) and 60% having iris abnormalities. Vitreous haemorrhage was present in 66% with three-quarters of these being fundus obscuring. Approximately half of all patients had retinal detachment at the time of vitrectomy surgery with PVR already present in half of these. However, all patients in ASCOT were listed for vitrectomy, rather than being observed. The rapid development of PVR is notable, as the mean interval between injury and surgery was 20 days. The fovea was detached in over half (60%) of detachments.

In the largest retrospective review to date of open globe injuries (848 patients), Andreoli et al. report that approximately 1/3 of patients (29%) proceed to vitrectomy surgery [23]. In a subsequent report of an expanded cohort of 894 open globe injury patients, retinal detachment complicated 29% of patients. Vitreous haemorrhage was significantly associated with the presence of retinal detachment where 85% of eyes with retinal detachment had an associated vitreous haemorrhage compared with a 32% incidence of vitreous haemorrhage in eyes without retinal detachment (*p* ≤ 0.001). Additionally, they reported a significant higher risk of retinal detachment in older, male patients with poorer presenting vision and higher zone of injury (Zone 3, Scleral posterior to muscle insertion) [24].

Endophthalmitis was rare (2%) on presentation. Pre-existing eye disease was uncommon: four of the 280 patients had glaucoma and one had had previous macular disease recorded—this may reflect the younger age range of patients sustaining open globe eye trauma.

The presenting clinical characteristics which were associated with better presenting vision were often related to ocular media opacities. The presence of corneal scarring, cataract, hyphaema and vitreous haemorrhage conferred less good presenting vision. Eyes which had previously undergone surgery also had less good vision possibly relating to more complex pre-existing intraocular pathology. Eyes with ACIOLs had better visual acuity – this may reflect a more longstanding and stable situation prior to the need for vitreoretinal intervention, however, this should be interpreted with caution as there were only two patients with ACIOLs. The presence of PVR (and by implication retinal detachment) on presentation was also associated with less good presenting vision. PVR is associated with poor vision and poor outcomes from surgery both with and without previous ocular trauma [9, 25].

The associations with retinal detachment at presentation are also notable. The zone of injury clearly influences the likelihood of retinal and vitreous involvement in the injury and as expected more posterior injuries were more likely to have retinal detachment on presentation. The association with hyphaema could potentially reflect the potential of intraocular blood to promote PVR through fibrogenic growth factors.

Recruitment for the study was centred in London, particularly Moorfields Eye Hospital which was the lead site. The patient cohort is therefore not a comprehensive study of open globe injury in the UK. To compare regions, cases were grouped together to provide cohorts of a meaningful size for analysis. The cases recruited in London were generally less severe (a greater proportion of corneal, zone 1 (cornea), injuries) than elsewhere with more ocular co-morbidities. These observations must, however, be treated with caution as they may be influenced by recruitment bias in the trial centres.

There was a trend for recruitment to be higher in spring (March and April) and October although this was not statistically significant with the patient numbers in the study. Notably workplace injuries had a trend to more common in spring and summer with interpersonal violence being more common in January and December. It is possible that a reduction in work and an increase in in social interaction results in the reversal of causation seen in December and January. Although a bias in case recruitment may influence the monthly cases numbers overall, it is unlikely to have affected the variation in injury causation seen.

## Conclusion

The ASCOT study provides detailed clinical data on an extensive cohort of patients with open globe penetrating injuries undergoing vitreoretinal surgery. Although not a comprehensive epidemiological study, this study provides valuable insights into the spectrum of pathology encountered by vitreoretinal surgeons. We have documented the common settings for injury—in the workplace and through interpersonal violence, highlighted a trend in seasonal variation and reported the clinical presentations of the cases—predominantly penetrating or globe rupture and often with media opacities limiting the fundal view. These observations can help training and in planning the resources needed to deal with often challenging surgical cases.

## Summary

### What was known before

- Ocular trauma is a leading cause of monocular visual impairment and blindness worldwide, affecting 55 million people every year.
- Ocular trauma is a serious health problem that has extensive, variable, physical and psychological impacts on patients and their relatives.
- The primary aim of the Adjunctive Steroid Combination in Ocular Trauma (ASCOT) trial is to assess whether, adjunctive intravitreal and sub-Tenon’s triamcinolone acetonide given at the time of surgery improves visual acuity at 6 months compared with standard surgery.

### What this study adds

- Common settings for open globe injury for the ASCOT cohort include the workplace and through interpersonal violence, with a trend in seasonal variation.
- There is considerable variability in presenting visual acuity for patients with open globe eye injuries undergoing vitreoretinal surgery.
- Clinical presentations of cases are predominantly penetrating or globe rupture and often with media opacities limiting the fundal view.

## Supplementary information

Presenting Clinical Characteristics of Open Globe Injuries in Ocular Trauma: Baseline Analysis of Cases in the ASCOT National Clinical Trial - Supplementary file 1

## Data Availability

The datasets generated and analysed during the current study are available from the corresponding author on reasonable request.
