# Supplementary material for: Presenting clinical characteristics of open globe injuries in ocular trauma: baseline analysis of cases in the ASCOT national clinical trial
Source: Eye (Lond). 2022 Sep 14;37(8):1732–40. doi: 10.1038/s41433-022-02206-z (PMC10220025; doi:10.1038/s41433-022-02206-z)
Supplement: Supplementary file 1 — Presenting Clinical Characteristics of Open Globe Injuries in Ocular Trauma: Baseline Analysis of Cases in the ASCOT National Clinical Trial - Supplementary file 1 [file 41433_2022_2206_MOESM1_ESM.docx]

**Presenting Clinical Characteristics of Open Globe Injuries in Ocular Trauma: Baseline Analysis of Cases in the ASCOT National Clinical Trial - Supplementary file 1**

| **Investigator** | **Site** |
| --- | --- |
| Mr Arijit Mitra | Birmingham and Midland Eye Centre (BMEC). |
| Prof Tim Jackson | Kings College Hospital London |
| Mr Luke Membrey | Maidstone and Tunbridge Wells NHS Trust |
| Mr Jonathan Smith | Sunderland Eye Infirmary |
| Ms Arabella Poulson | Addenbrookes Hospital , Cambridge |
| Prof Ian Pearce | St Pauls Eye Unit , Liverpool |
| Mr Felipe Dhawahir-Scala | Manchester Royal Eye Hospital |
| Mr Yash Ramkissoon | Royal Hallamshire Hospital, Sheffield |
| Mr Alistair Laidlaw | St Thomas’ Hospital London |
| Ms Daniela Vaideanu-Collins | South Tees Hospital NHS Trust |
| Mr Mark Costen | Hull University Teaching Hospitals |
| Ms Cordelia McKechnie | Whipps Cross Hospital London |
| Mr Richard Haynes | University Hospitals Bristol |
| Dr Jas Singh | Princess Alexandra Eye Pavilion, Edinburgh |
| Dr David Yorston | Tennent Institute of Ophthalmology, Glasgow |
| Ms Rahila Zakir | Western Eye Hospital London |
| Mr Fung Yang | Queen Alexandra Hospital Portsmouth |
| Prof Robert MacLaren | Oxford Eye Hospital |
| Mr Fred Frimpong-Ansah | Royal Eye Infirmary Plymouth |
| Mr Ravikiran Gandhewa | University Hospital, Derby |
| Mr Aman Chandra | Mid and South Essex NHS Foundation Trust |
| Mr Kam Balaggan | Royal Wolverhampton NHS Trust |
| Ms Roxane Hillier | Newcastle Upon Tyne NHS Foundation Trust |
| Mr David Schultz | East Kent Hospital NHS Foundation Trust |
| Mr Mandeep Bindra | Stoke Mandeville Hospital |
| Mr Phil Banerjee | Frimley Park Hospital, NHS Foundation Trust |
| Prof David Charteris | Moorfields Eye Hospital London |

## Principal Investigators for ASCOT

## Other Injury causes

Injury causes were recorded as workplace accidents, road traffic accidents, interpersonal violence, sports injury, domestic gardening, domestic diy, fall, other domestic and other injury. For injuries classified as other injury and other domestic we list the additional details captured for these events below.

Table S1a - Other injury details (n=37):

| **No. of injuries** | **Cause of other injury** |
| --- | --- |
| 5 | accident (no other description) |
| 3 | unknown |
| 2 | recreational hobby (no other description) |
| 2 | leisure as child |
| 2 | accident - elbow |
| 1 | eye hit by sword during acting |
| 1 | hammmering metal against roof tile , fb in eye. |
| 1 | accidental playing with friends |
| 1 | recreational hobby( motel helicopter) |
| 1 | iofb |
| 1 | stabbed himself with a penknife |
| 1 | airgun pillit |
| 1 | hunting accident |
| 1 | hit by branch |
| 1 | hit by car door accidentally |
| 1 | chopping wood |
| 1 | hit by a piece of metal accidently |
| 1 | watching someone hammer nail |
| 1 | accidentally poked by sibling with a stick |
| 1 | hammering metal |
| 1 | firework injury |
| 1 | festival |
| 1 | stick in the eye |
| 1 | self inflicted |
| 1 | with glass |
| 1 | non work incident |
| 1 | personal accident while under influence |
| 1 | accident, tripped over |

Table S1b - Other domestic injury (n=21):

| **No. of injuries** | **Cause of domestic injury** |
| --- | --- |
| 7 | home accident (no other description) |
| 3 | home injury |
| 3 | at home (no other description) |
| 1 | accidental injury with husband's elbow |
| 1 | accidental event (parasol end vs left eye) |
| 1 | home accident at friend's |
| 1 | child opened door at home - hit the right eye |
| 1 | oven door injury |
| 1 | using a knife in the kitchen |
| 1 | hit eye on arm of a chair |
| 1 | home. injury occurred with a utensil ( fork) |

## Baseline characteristics by region

Table S2 - Baseline demographics, trauma history and ocular history by region

| **Baseline Demographics by Region [n missing]** | **London** | **Southern England** | **Midlands + North England** | **Scotland** |
| --- | --- | --- | --- | --- |
| Gender (n, %) [n=0] |  |  |  |  |
| Female | 22 (15%) | 6 (11%) | 6 (9%) | 0 (0%) |
| Male | 126 (85%) | 49 (89%) | 64 (91%) | 7 (100%) |
| Ethnicity (n, %) [n=0] |  |  |  |  |
| White | 111 (75%) | 53 (96%) | 62 (89%) | 7 (100%) |
| Black | 17 (11%) | 1 (2%) | 2 (3%) | 0 (0%) |
| Asian | 14 (9%) | 1 (2%) | 3 (4%) | 0 (0%) |
| Other | 5 (3%) | 0 (0%) | 1 (1%) | 0 (0%) |
| Mixed | 1 (1%) | 0 (0%) | 2 (3%) | 0 (0%) |
| Eye injured (n, %) [n=0] |  |  |  |  |
| Right | 71 (48%) | 31 (56%) | 34 (49%) | 1 (14%) |
| Left | 73 (49%) | 24 (44%) | 35 (50%) | 6 (86%) |
| Both | 4 (3%) | 0 (0%) | 1 (1%) | 0 (0%) |
| How was the eye injured (n, %) [n=0] |  |  |  |  |
| Workplace incident | 43 (29%) | 17 (31%) | 24 (34%) | 4 (57%) |
| Road traffic accident | 5 (3%) | 4 (7%) | 2 (3%) | 0 (0%) |
| Interpersonal violence | 37 (25%) | 11 (20%) | 17 (24%) | 1 (14%) |
| Sports injury | 5 (3%) | 4 (7%) | 1 (1%) | 0 (0%) |
| Other Injury* | 23 (16%) | 4 (7%) | 10 (14%) | 0 (0%) |
| Other Domestic^†^ | 11 (7%) | 3 (5%) | 7 (10%) | 0 (0%) |
| Domestic Gardening | 4 (3%) | 1 (2%) | 3 (4%) | 0 (0%) |
| Domestic DIY | 6 (4%) | 3 (5%) | 3 (4%) | 1 (14%) |
| Iatrogenic | 2 (1%) | 0 (0%) | 1 (1%) | 0 (0%) |
| Fall | 12 (8%) | 8 (15%) | 2 (3%) | 0 (0%) |
| Was there previous primary repair? - Yes (n, %) [n=1] | 110 (75%) | 44 (80%) | 47 (67%) | 4 (57%) |
| Severity of trauma: classification (n, %) [n=0] |  |  |  |  |
| Rupture | 46 (31%) | 33 (60%) | 30 (43%) | 4 (57%) |
| Penetrating | 70 (47%) | 14 (25%) | 19 (27%) | 0 (0%) |
| Perforating | 6 (4%) | 3 (5%) | 2 (3%) | 0 (0%) |
| IOFB | 26 (18%) | 5 (9%) | 19 (27%) | 3 (43%) |
| Severity of trauma: extent (n, %) [n=3] |  |  |  |  |
| Cornea | 58 (39%) | 15 (27%) | 19 (28%) | 3 (43%) |
| Scleral anterior to muscle insertion | 52 (35%) | 21 (38%) | 33 (49%) | 4 (57%) |
| Scleral posterior to muscle insertion | 37 (25%) | 19 (35%) | 16 (24%) | 0 (0%) |
| Severity of trauma: RAPD present? - Yes (n, %) [n=2] | 13 (9%) | 11 (20%) | 19 (28%) | 0 (0%) |
| Not documented | 82 | 29 | 26 | 3 |
| Glaucoma – Yes (n, %) [n=1] | 2 (1%) | 1 (2%) | 1 (1%) | 0 (0%) |
| Previous eye surgery (study eye) – Yes (n, %) [n=0] | 101 (68%) | 13 (24%) | 31 (44%) | 4 (57%) |
| Macular disease – Yes (n, %) [n=1] | 0 (0%) | 0 (0%) | 1 (1%) | 0 (0%) |
| Other historic ocular conditions- Yes (n, %) [n=2] | 21 (14%) | 1 (2%) | 7 (10%) | 0 (0%) |
| Visual axis corneal scar – Yes (n, %) [n=0] | 41 (28%) | 12 (22%) | 18 (26%) | 1 (14%) |
| Uveitis – Yes (n, %) [n=0] | 20 (14%) | 14 (25%) | 15 (21%) | 3 (43%) |
| Hyphaemia level (n, %) [n=0] |  |  |  |  |
| No hyphaemia present | 113 (76%) | 31 (56%) | 37 (53%) | 5 (71%) |
| <50% | 23 (16%) | 10 (18%) | 16 (23%) | 1 (14%) |
| >50% | 12 (8%) | 14 (25%) | 17 (24%) | 1 (14%) |
| Iris state (n, %) [n=4] |  |  |  |  |
| Normal | 61 (41%) | 17 (31%) | 29 (42%) | 4 (57%) |
| Incomplete | 74 (50%) | 30 (56%) | 30 (43%) | 1 (14%) |
| Incarcerated | 12 (8%) | 7 (13%) | 9 (13%) | 2 (29%) |
| Lens state (n, %) [n=3] |  |  |  |  |
| Clear | 35 (24%) | 8 (15%) | 23 (34%) | 4 (57%) |
| Cataract | 46 (31%) | 21 (39%) | 27 (40%) | 2 (29%) |
| Aciol | 1 (1%) | 1 (2%) | 0 (0%) | 0 (0%) |
| Pciol | 17 (11%) | 1 (2%) | 2 (3%) | 0 (0%) |
| Aphakic | 49 (33%) | 23 (43%) | 16 (24%) | 1 (14%) |
| Vitreous haemorrhage present – Yes (n, %) [n=4] | 82 (55%) | 43 (81%) | 53 (78%) | 4 (57%) |
| If vitreous haemorrhage... (n, %) [n=0] |  |  |  |  |
| No fundal view | 64 (78%) | 35 (81%) | 38 (72%) | 2 (50%) |
| VH with fundus visible | 18 (22%) | 8 (19%) | 15 (28%) | 2 (50%) |
| Endophthalmitis present – Yes (n, %) [n=1] | 4 (3%) | 1 (2%) | 0 (0%) | 0 (0%) |
| Retinal status (n, %) [n=0] |  |  |  |  |
| Attached | 68 (46%) | 30 (55%) | 33 (47%) | 6 (68%) |
| TRD | 19 (13%) | 7 (13%) | 12 (17%) | 0 (0%) |
| RRD | 61 (41%) | 18 (33%) | 25 (36%) | 1 (14%) |
| Was the fovea off – Yes (n, %) [n=0] | 49 (61%) | 17 (68%) | 18 (49%) | 1 (100%) |
| Splitting | 1 (1%) | 0 (0%) | 0 (0%) | 0 (0%) |
| Was PVR present – Yes (n, %) [n=1] | 43 (29%) | 8 (15%) | 16 (23%) | 0 (0%) |
| Age (years) [n=0] |  |  |  |  |
| Median, IQR | 41.5 (29.3, 56.0) | 50.3 (33.7, 57.1) | 43.4 (33.3, 52.9) | 38.8 (35.4, 60.7) |
| Time from injury to surgery (days) [n=0] |  |  |  |  |
| Median, IQR | 30.5(12.0, 139.0) | 16.0 (7.0, 33.0) | 13.0 (3.0, 27.0) | 12.0 (1.0, 17.0) |
| IOP in eye operated on [n=26] |  |  |  |  |
| Median, IQR | 10.0 (8.0, 14.0) | 14.0 (8.0, 16.0) | 10.0 (7.0, 18.0) | 12.0 (11.0, 16.0) |
| Categorised IOP [n=26] |  |  |  |  |
| Low IOP (<6) | 21 (15%) | 6 (12%) | 11 (19%) | 1 (20%) |
| Normal IOP (6<=x<=22) | 107 (77%) | 40 (78%) | 44 (75%) | 4 (80%) |
| High IOP (>22) | 11 (8%) | 5 (10%) | 4 (7%) | 0 (0%) |
| ETDRS in eye operated on (total score) [n=0] |  |  |  |  |
| Mean, SD | 15.5 (28.3) | 9.1 (24.4) | 11.7 (26.7) | 20.7 (33.6) |
| Median, IQR | 0.0 (0.0, 16.0) | 0.0 (0.0, 0.0) | 0.0 (0.0, 0.0) | 0.0 (0.0, 45.0) |
| Min, Max | 0.0 (0.0, 93.0) | 0.0 (0.0, 88.0) | 0.0 (0.0, 100.0) | 0.0 (0.0, 87.0) |
| EDTRS score in eye operated on [n=0] |  |  |  |  |
| 0 | 101 (68%) | 48 (87%) | 56 (80%) | 4 (57%) |
| >0 | 47 (32%) | 7 (13%) | 14 (20%) | 3 (43%) |
| Where ETDRS=0, level of vision in eye operated on[n=0] |  |  |  |  |
| Counting finger | 7 (7%) | 2 (4%) | 8 (14%) | 2 (50%) |
| Hand movement | 63 (62%) | 25 (52%) | 25 (45%) | 1 (25%) |
| Perception of light | 30 (30%) | 19 (40%) | 21 (38%) | 1 (25%) |
| No perception of light | 1 (1%) | 2 (4%) | 2 (4%) | 0 (0%) |
| Where ETDRS>0 in eye operated on [n=0] |  |  |  |  |
| Mean, SD | 49.0 (29.9) | 71.3 (13.3) | 58.5 (28.9) | 48.3 (37.1) |
| Median, IQR | 51 (19, 80) | 73 (58, 83) | 60 (34, 75) | 45 (13, 87) |
| Min, Max | (1, 93) | (53, 88) | (5, 100) | (13, 87) |

*Note: Percentages have been rounded throughout to nearest 1 d.p. so may not always sum to 100%*

** Other Injury includes any injuries that do not fall into any other category (e.g. freak accidents/childhood play), or where not enough information was given to categorise*

*^†^ Other Domestic includes all other injuries within a domestic setting not covered by Domestic Gardening, Domestic DIY or Falls*

*IOFB=Intraocular foreign body. TRD=Tractional retinal detachment, RRD=* *rhegmatogenous retinal detachment. VH= Vitreous haemorrhage. RAPD= Relative Afferent Pupillary Defect. PVR= Proliferative vitreoretinopathy. IOP=Intraocular pressure.*

## Associations between trauma and ocular history and presenting vision – additional methods and results

The selected clinically important characteristics that were of interest to explore the association with presenting vision in the ASCOT cohort were:

previous primary repair, trauma classification, extent of trauma, previous eye surgery, macular disease, other historical ocular affliction, visual axial corneal scarring, hyphaemia, lens state, vitreous haemorrhage, endophtamlitis, retinal status and PVR.

Since approximately 75% of all participants had an ETDRS total score of 0 (see Figure S1) a zero inflated negative binomial model was used to explore the associations between trauma and ocular history and presenting vision. This model is appropriate for count data that has a high fraction of zeros and consists of a logistic model to model zero/very low vision (ETDRS = 0) versus ETDRS >0 and a negative binomial model to explain the vision score where ETDRS >0.

Initially we attempted to fit the model with all the clinically important variables (see table 3) in both the zero part of the model and the negative binomial model. However the model did not converge. We therefore fitted a separate logistic model to first identify the variables associated with zero/very low vision (see Table S1). The variables that had a significant association with having zero/very low vision within the binomial model (p<0.2) were then used in the zero inflated model. These included visual axis corneal scar, hyphaemia, vitreous haemorrhage and proliferative vitreoretinopathy. The negative binomial component of the model included all the clinically important variables.

Figure S1 – Presenting ETDRS scores

Table S3 – Associations with presenting zero/very low vision (ETDRS = 0) versus ETDRS>0

| **Variable** | **OR*** | **95% CI** | **p-value** |
| --- | --- | --- | --- |
| Previous primary repair |  |  |  |
| No (reference) |  |  |  |
| Yes | 0.87 | 0.24, 3.09 | 0.826 |
| Classification |  |  |  |
| Rupture (reference) |  |  | 0.684 |
| Penetration | 0.62 | 0.22, 1.80 |  |
| Perforation | 2.22 | 0.11, 45.87 |  |
| IOFB | 0.58 | 0.15, 2.23 |  |
| Extension zone |  |  |  |
| Cornea (reference) |  |  | 0.634 |
| Scleral – anterior to muscle insertion | 0.62 | 0.22, 1.74 |  |
| Scleral – posterior to muscle insertion | 0.84 | 0.20, 3.57 |  |
|  |  |  |  |
| Previous eye surgery (eye operating on) |  |  |  |
| No (reference) |  |  |  |
| Yes | 1.41 | 0.46, 4.32 | 0.543 |
| Macular disease |  |  |  |
| No (reference) |  |  |  |
| Yes | N/A |  |  |
| Other eye history |  |  |  |
| No (reference) |  |  |  |
| Yes | 0.80 | 0.24, 2.66 | 0.719 |
| Visual axis corneal scar |  |  |  |
| No (reference) |  |  |  |
| Yes | 2.05 | 0.74, 5.63 | 0.165 |
| Hyphaemia |  |  |  |
| None (reference) |  |  | 0.126 |
| <50% | 3.19 | 0.75, 13.65 |  |
| >50% | 5.46 | 0.55, 53.78 |  |
| Lens status |  |  |  |
| Clear (reference) |  |  | 0.161 |
| Cataract | 2.83 | 0.92, 8.64 |  |
| Aciol | N/A |  |  |
| Pciol | 1.00 | 0.20, 4.95 |  |
| Aphakic | 2.36 | 0.68, 8.19 |  |
| Vitreous haemorrhage |  |  |  |
| None (reference) |  |  | <0.001 |
| VH with no fundal view | 9.00 | 2.76, 29.34 |  |
| VH with fundus | 0.36 | 0.12, 1.06 |  |
| Endophtamlitis |  |  |  |
| No (reference) |  |  | 0.648 |
| Yes | 0.56 | 0.05, 6.73 |  |
| Retinal status |  |  |  |
| Attached (reference) |  |  | 0.833 |
| TRD | 1.81 | 0.27, 12.28 |  |
| RRD | 1.08 | 0.46, 2.56 |  |
| Proliferative vitreoretinopathy (PVR) |  |  |  |
| No (reference) |  |  | 0.014 |
| Yes | 5.35 | 1.41, 20.36 |  |

†OR (Odds ratio) represents the odds of ETRDS score <=0, for the associated variable relative to the reference group for that variable. *IOFB=Intraocular foreign body.* *TRD=Tractional retinal detachment, RRD=* *rhegmatogenous retinal detachment. VH= Vitreous haemorrhage. PVR= Proliferative vitreoretinopathy.*
